# Lack of PPAR*β*/*δ*-Inactivated SGK-1 Is Implicated in Liver Carcinogenesis

**DOI:** 10.1155/2020/9563851

**Published:** 2020-10-02

**Authors:** Bo Shen, Aimin Li, Yu-Jui Yvonne Wan, Guijia Shen, Jinshui Zhu, Yuqiang Nie

**Affiliations:** ^1^Department of Gastroenterology, Shanghai Jiao Tong University Affiliated Shanghai Sixth Hospital, Shanghai, China; ^2^Department of Gastroenterology and Hepatology, Guangzhou First Municipal People's Hospital, Guangzhou Medical University, Guangzhou, China; ^3^Guangdong Provincial Key Laboratory of Gastroenterology, Nanfang Hospital, Southern Medical University, Guangzhou, China; ^4^Department of Pathology and Laboratory Medicine, University of California, Davis, Sacramento, CA, USA; ^5^Department of Gastroenterology and Hepatology, The Affiliated Hospital of Jiujiang, Jiangxi, China

## Abstract

**Objective:**

The present study examined the role of PPAR*β*/*δ* in hepatocellular carcinoma (HCC).

**Methods:**

The effect of PPAR*β*/*δ* on HCC development was analyzed using PPAR*β*/*δ*-overexpressed liver cancer cells and PPAR*β*/*δ*-knockout mouse models.

**Results:**

PPAR*β*/*δ*^(-/-)^ mice were susceptible to diethylnitrosamine- (DEN-) induced HCC (87.5% vs. 37.5%, *p* < 0.05). In addition, PPAR*β*/*δ*-overexpressed HepG2 cells had reduced proliferation, migration, and invasion capabilities accompanied by increased apoptosis and cell cycle arrest at the G0/G1 phase. Moreover, differential gene expression profiling uncovered that the levels of serine/threonine-protein kinase (SGK-1) mRNA and its encoded protein were reduced in PPAR*β*/*δ*-overexpressed HepG2 cells. Consistently, elevated SGK-1 levels were found in PPAR*β*/*δ*^(-/-)^ mouse livers as well as PPAR*β*/*δ*-knockdown human SMMC-7721 HCC cells. Chromatin immunoprecipitation (ChIP) assays followed by real-time quantitative polymerase chain reaction (qPCR) assays further revealed the binding of PPAR*β*/*δ* to the SGK-1 regulatory region in HepG2 cells.

**Conclusions:**

Due to the known tumor-promoting effect of SGK1, the present data suggest that PPAR*β*/*δ*-deactivated SGK1 is a novel pathway for inhibiting liver carcinogenesis.

## 1. Introduction

Peroxisome proliferator-activated receptors (PPARs) are ligand-activated transcription factors, of which three isoforms exist: *α*, *γ*, and *β*/*δ* [1–3]. PPAR*β*/*δ* is the most widely expressed member of the PPAR family in human tissues and is abundantly found in the skin, intestine, and liver [4, 5]. PPAR*β*/*δ* is implicated in differentiation [6, 7], anti-inflammation [8], fatty acid catabolism [9], and preventing interleukin-6- (IL-6-) induced insulin resistance [10]. In animal models, PPAR*β*/*δ* agonists attenuate hepatic steatosis by enhancing fatty acid oxidation, reducing lipogenesis, and improving insulin sensitivity [ 11]. In humans, PPAR*β*/*δ* agonists reduce the hepatic fat content and elicit improvements in the plasma markers of liver function [12]. Furthermore, PPAR*β*/*δ* activation and overexpression inhibit lipogenesis in hepatocytes by increasing the expression of insulin-induced gene-1 **[**13].

Hepatocellular carcinoma (HCC) is one of the deadliest forms of cancer, and very limited data are available on the role of PPAR*β*/*δ* in HCC development. Studies have indicated that PPAR*β*/*δ* is a feasible target for chemoprevention in the last 10 years [14], although the functional outcomes of PPAR*β*/*δ* activation in some cancers are contradictory [15, 16]. However, the Human Protein Atlas database indicates that PPAR*β*/*δ* is undetectable in 80% of HCCs [14]. Nevertheless, it has been shown that PPAR*β*/*δ* activation promotes the proliferation and growth of human hepatic cancer cell lines through the upregulation of cyclooxygenase-2 (COX-2) and prostaglandin E_2_ production [17]. In contrast, another study has demonstrated that the COX-2 expression was not affected when human HCC cell lines were treated with PPAR*β*/*δ* ligands [18]. Therefore, the role of PPAR*β*/*δ* in hepatocarcinogenesis warrants further investigation. The aim of this study was to investigate the functional significance of PPAR*β*/*δ* in liver cancer cells and mouse models. Our data revealed the anti-HCC effect of PPAR*β*/*δ* and that PPAR*β*/*δ*-regulated serine/threonine-protein kinase (SGK-1) is implicated in the anti-HCC effect. In summary, PPAR*β*/*δ*-deactivated SGK-1 is a novel pathway for inhibiting tumor growth and linking metabolism and liver carcinogenesis together.

## 2. Materials and Methods

### 2.1. Experimental Animals and Study Design

PPAR*β*/*δ*-null mice in the C57BL/6 background were provided by Dr. Frank J. Gonzalez at the National Cancer Institute, National Institutes of Health, Bethesda, MD [19]. Genotyping was confirmed using the polymerase chain reaction (PCR), and animals were housed under controlled temperature (21 ± 1°C) conditions with a 12 h light-dark cycle and were allowed free access to food and water. Wild-type or PPAR*β*/*δ*-null mice (male, 15 days old; 8 per group) were given a single intraperitoneal injection of diethylnitrosamine (DEN) (5 mg/kg body weight; Sigma Chemical Co., St. Louis, MO) [19]. The mice were anesthetized by chloroform and were sacrificed without fasting at the indicated time points. Blood was collected by cardiac puncture, and the livers were excised and weighed. The presence and dimensions of the surface nodules were evaluated and recorded. Each liver was cut into strips of 2–3 mm in thickness to examine the presence of macroscopically visible lesions. HCC was diagnosed by an experienced pathologist based on gross or histological examination. All of the animal experiments were conducted in accordance with the guidelines provided by the Animal Experimentation Ethics Committee of Guangzhou Medical University.

### 2.2. Human Liver Cancer Cell Culture

Five liver cancer cell lines, HepG2, Huh7, Hep3B, SMMC7721 (ATCC, Manassas, VA), and MHCC97H (Shanghai Institute of Biochemistry and Cell Biology, Shanghai, China), were maintained in Dulbecco's modified Eagle's medium, supplemented with 10% fetal bovine serum (FBS) and 1% penicillin-streptomycin (Gibco, Gaithersburg, MD).

### 2.3. PPAR*β*/*δ* Expression and Transfection

The pEGFP-PPAR*β*/*δ* and pEGFP vectors were constructed by Genechem Co., Ltd. (Shanghai, China) and were used for transfection by Lipofectamine 2000 (Invitrogen, Carlsbad, CA). PPAR*β*/*δ*-overexpressed HepG2 cells were selected using 800 *μ*g/mL G418 (Mpbio) after transfection for 48 h. The cell lines were named as HepG2_PPAR*β*/*δ* and HepG2_mock, respectively.

### 2.4. RNA Interference and Transfection

The SMMC-7721-NC and SMMC-7721-shPPARD cells were generated using lentiviral transduction of LV008-shPPAR*β*/*δ* (shPPARD) or control LV008 vectors (NC) (Forevergen. China) into SMMC-7721 cells, respectively, followed by selection of stable cell lines in puromycin (2 *μ*g/mL). The sequence of shPPARD was 5′-AACT CAGTGATATCATTGAGCCTAATTCAAGAGATTAGGCTCAATGATATCACGTTTTTTC-3′.

### 2.5. RNA Extraction and Real-Time Quantitative PCR (qPCR)

Total RNA was extracted using TRIzol (Invitrogen, Carlsbad, CA) and reverse-transcribed with oligo (dT) and M-MLV reverse transcriptase (Invitrogen). qPCR was performed with the GoTaq® qPCR Master Mix kit (Promega, A6002). The primer pairs were designed with Primer Premier 5, and the sequences were as follows: PPAR*β*, F 5′-GGGCTTCCACTACGGTGTTCAT-3′, R 5′-TACTGGCACTTGTTGCGGTTCTT-3′; SGK-1, F 5′-CAAATAGAGGTTCAAGGGAT-3′, R 5′-TTAGGAGGCTTAGGTGGA-3′; and glyceraldehyde-3-phosphate dehydrogenase (GAPDH), F 5′-GAGTCAACGGATTTGGTCGT-3′, R 5′-GACAAGCTTCCCGTTCTCAG-3′. GAPDH was used to normalize the mRNA level.

### 2.6. Western Blotting

The cells were washed and lysed, and the clarified lysates were processed for western blot analysis. The extracted protein sample was separated by sodium dodecyl sulfate–polyacrylamide gel electrophoresis and transferred onto polyvinylidene difluoride membranes. The blots were blocked with 5% nonfat milk and incubated with specific primary antibodies against PPAR*β*/*δ* (1 : 500, Santa Cruz), SGK-1 (1 : 2000, Abcam, Cambridge, MA), and GAPDH (1 : 5000, Abcam). The proteins were then incubated with the secondary antibody (1 : 2000, Abcam) and detected by enhanced chemiluminescence (Amersham Corp., UK).

### 2.7. Immunohistochemical Analysis of SGK-1

The paraffin-embedded liver sections of PPAR*β*^(-/-)^ and wild-type mice were analyzed by immunohistochemistry using the monoclonal antibody specific for SGK-1 (1 : 200, Abcam). Positive signals were visualized by diaminobenzidine and counterstained with hematoxylin. The immunostaining intensity was scored by an experienced pathologist as follows: 0, no staining; 1, mild staining; 2, moderate staining; and 3, strong staining. The percentage of positive cells was semiquantitatively scored as follows: 0, <5%; 1, 6–25%; 2, 26–50%; 3, 51–75%; and 4, >75%. The final immunoreactivity score was calculated by adding the intensity and percentage scores.

### 2.8. Colony Formation Assay

HepG2 cells were transfected with GV230-PPAR*β*/*δ* or an empty vector to the preseeded cells in 6-well plates at a density of 50, 100, or 200 cells per well. After 14 days of stationary culture, the cells were fixed with 70% ethanol and stained with crystal violet (Sigma, St. Louis, MO). Colonies with more than 50 cells/colony were counted under a microscope to calculate the rate of colony formation. All of the data were obtained from three independent experiments.

### 2.9. Cell Growth Assay

The cell viability of HepG2_PPAR*β*/*δ* and HepG2_mock cells was determined by the cell counting kit-8 (CCK-8; Beyotime) in a 96-well plate at a density of 1 × 10^4^ cells/well. The optical density was measured at different time points.

### 2.10. Cell Cycle and Apoptosis Analysis

Flow cytometry was used to observe the cell cycle distribution and apoptosis. HepG2_PPAR*β*/*δ* and HepG2_mock cells were incubated with 10% FBS for 24 h after a serum starvation period of 12 h. The cells were fixed in 70% ethanol and stained with 50 *μ*g/mL propidium iodide (BD Pharmingen, San Jose, CA). Then, the cells were sorted by FACSCalibur (BD Biosciences, San Jose, CA), and the cell-cycle profiles were analyzed by the Flowjo software (Leonard A. Herzenberg, Stanford University, Palo Alto, CA). For apoptosis examination, HepG2_PPAR*β*/*δ* and HepG2_mock cells were stained with fluorescein isothiocyanate- (FITC-) conjugated annexin V and 7-amino-actinomycin, according to the manufacturer's instructions (BD Biosciences).

### 2.11. Migration and Invasion Assays

The wound-healing assay was performed *in vitro* for cell migration analysis. Briefly, HepG2_PPAR*β*/*δ* and HepG2_mock cells (5 × 10^5^ cells/well) were cultured in 6-well plates until they reached 90% confluency [20]. Sterile tips were used to scratch the cell layers. Images of the wound closure areas were taken at 0, 24, and 48 h.

Matrigel migration and invasion assays were performed on HepG2_PPAR*β*/*δ* and HepG2_mock stably transfected liver cancer cells using 24-well Matrigel-biocoated migration and invasion chambers (Becton Dickinson, Waltham, MA), as previously described [21].

### 2.12. Microarray Analysis

The gene expression profiles of PPAR*β*/*δ*-overexpressed and empty vector-treated cells were obtained by oligonucleotide microarray analysis using an Illumina kit, according to the manufacturer's instructions. Data were collected using the Illumina Genome Studio software. Functional annotation was carried out using gene lists submitted to a variety of online software tools, including the Database for Annotation, Visualization and Integrated Discovery (DAVID) [22] and Gene Set Enrichment Analysis (GSEA) [23].

### 2.13. Chromatin Immunoprecipitation (ChIP) Assay

ChIP assays were performed on HepG2 cells transfected with pEGFP-PPAR*β*/*δ* or pEGFP vectors (used as a control) using an EZ-Magna ChIP A kit (Millipore, Billerica, MA). The cells were cross-linked with 1% formaldehyde (Sigma-Aldrich) for 10 min and quenched by glycine. The cross-linked cells were collected in cold phosphate-buffered saline and sonicated to reduce the total DNA size to 200–1000 bp. The chromatin DNA fragments were precipitated overnight with 10 *μ*g of PPAR*β*/*δ* antibody (Santa Cruz Biotechnology) or normal rabbit IgG at 4°C. The magnetic bead-antibody-chromatin complexes were washed, eluted, and incubated at 62°C for 2 h. The immunoprecipitated and input DNA was subjected to qPCR analysis using primers. The sequences of the SGK-1 promoter 1 were F 5′-CAAATAGAGGTTCAAGGGAT-3′ and R 5′-TTAGGAGGCTTAGGTGGA-3′.

## 3. Results

### 3.1. PPAR*β*/*δ* Deficiency Accelerates Hepatocarcinogenesis

**Figure 1:**
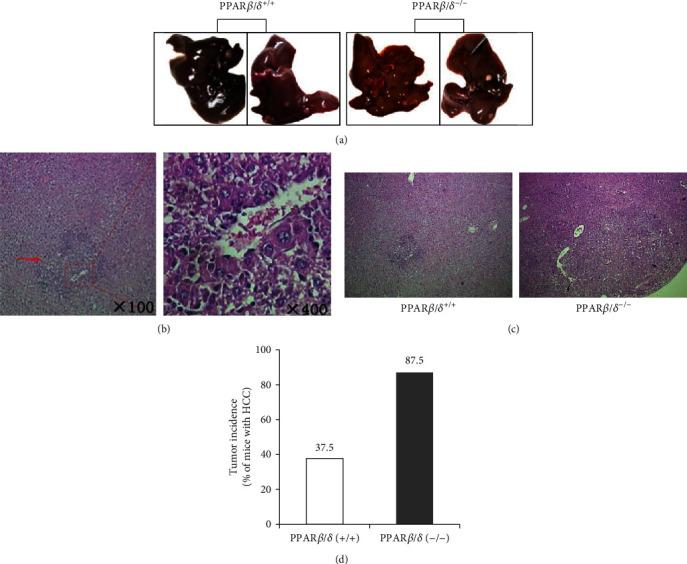
Role of PPAR*β*/*δ* in the upregulation of HCC. Mouse livers were excised after eight months of DEN treatment. (a) The photograph shows reduced tumor growth in the PPAR*β*/*δ*^(+/+)^ mice compared to the PPAR*β*/*δ*^(-/-)^ mice. (b) Hematoxylin-eosin-stained liver tissue sections of mice. (c) Representative histological results from HCC tissues showing HCC in hematoxylin-eosin-stained liver tissue sections of mice (magnification, 100x and 400x). Arrows indicate microscopic HCC. (d) Incidence of HCC development in PPAR*β*/*δ*^(+/+)^ and PPAR*β*/*δ*^(-/-)^ mice, which were kept under observation for eight months after the administration of DEN.

The mice developed HCC induced by DEN at 8 months. DEN induced HCC in 37.5% (3/8) of the wild-type mice, while the prevalence of HCC was much higher in the PPAR*β*/*δ*^(-/-)^ mice (87.5%, 7/8, *p* < 0.05). Moreover, the average number of tumors per animal was 2.8-fold higher in the PPAR*β*/*δ*^(-/-)^ mice compared with the wild-type mice (*p* < 0.05). Thus, PPAR*β*/*δ* deficiency increased the susceptibility of mice to DEN-induced hepatocarcinogenesis. No marked differences in the macroscopic or histological features of the HCCs were observed between the wild-type and PPAR*β*/*δ*-deficient mice, as evaluated by a pathologist (Figure 1).

### 3.2. Overexpression of PPAR*β*/*δ* Reduces Cell Proliferation and Induces Cell Cycle Arrest As Well As Apoptosis in HepG2 Cells

**Figure 2:**
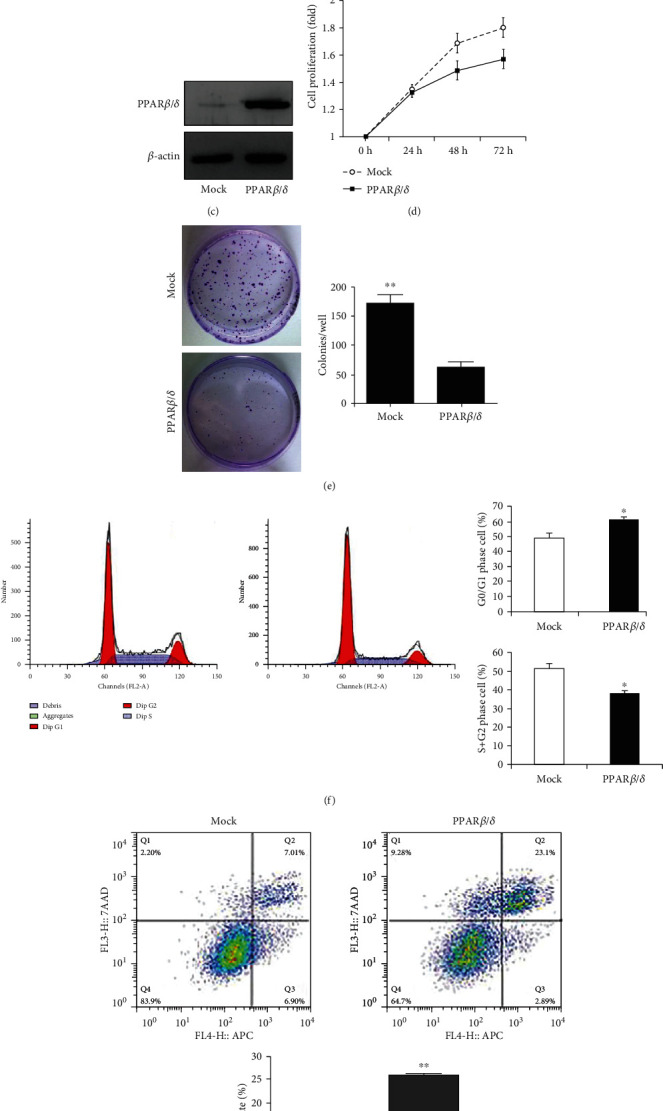
Effect of PPAR*β*/*δ* overexpression on cell growth, apoptosis, and cell cycle regulation. HepG2 cells were stably transfected with pEGFP-PPAR*β*/*δ* or pEGFP vector. (a) PPAR*β*/*δ* expression was analyzed in five different cell lines using western blot. (b) The relative mRNA expression levels for PPAR*β*/*δ* were evaluated by qPCR. The PPAR*β*/*δ* mRNA expression level was significantly higher in the PPAR*β*/*δ*-overexpressed cells than in the control cells (*p* < 0.001). (c) Western blotting analysis to evaluate the PPAR*β*/*δ* expression levels in HepG2 cells transfected with pEGFP-PPAR*β*/*δ* or the control vector. (d) Cell proliferation was assessed by the CCK-8 assay at the indicated time points. (e) The effect of PPAR*β*/*δ* on cancer cell growth was confirmed by a colony formation assay. Colonies were stained with 0.1% crystal violet and counted. (f) Representative histogram plots of the flow cytometry analysis. The numbers of cells in the G0/G1 and S+G2 phases were determined by flow cytometry. (g) The effect of PPAR*β*/*δ* on apoptosis was determined by FACS using an annexin V apoptosis assay. Annexin V-positive apoptotic cells were significantly increased in pEGFP-PPAR*β*/*δ*-transfected cells compared with pEGFP vector-transfected cells. Values are the mean of ± standard deviation from three replicate experiments. ^∗^*p* < 0.05, ^∗∗^*p* < 0.01.

An elevated PPAR*β*/*δ* protein level was observed in human HCC SMMC7721 cells, while HepG2 and MHCC97H cells did not express PPAR*β*/*δ* protein (Figure 2(a)). Therefore, HepG2 cells were used for PPAR*β*/*δ* overexpression, and overexpression was confirmed by qRT-PCR and western blotting in HepG2 cells transfected with pEGFP-PPAR*β*/*δ* (Figures 2(b) and 2(c)).

The effect of PPAR*β*/*δ* overexpression on the cell viability of HepG2 cells was analyzed by the CCK-8 assay. The enhanced PPAR*β*/*δ* expression suppressed the cell viability in a time-dependent fashion (Figure 2(d)). The suppressive effect on cancer cell growth was further confirmed by the colony formation assay in stably transfected cells. The colony numbers of pEGFP-PPAR*β*/*δ*-transfected cells were reduced to 38% of that of the control cells (*p* < 0.01; Figure 2(e)). To further characterize the influence of PPAR*β*/*δ* on cell growth, flow cytometry was used to analyze the cell cycle distribution in HepG2 cells transfected with pEGFP-PPAR*β*/*δ* or control pEGFP vectors. We found that the overexpression of PPAR*β*/*δ* in HepG2 cells resulted in significant inhibition of cell cycle progression and the accumulation of G0–G1 phase cells (61.7 ± 1.72% vs. 49.1 ± 3.2%, *p* < 0.05Figure 2(f)). Cell apoptosis was determined by annexin V–FITC/propidium iodide fluorescence-activated cell sorting (FACS) analysis. The results showed an increase in the number of early apoptotic cells (25.67 ± 0.531% vs. 13.71 ± 0.364%, *p* < 0.05) in HepG2 cells transfected with pEGFP-PPAR*β*/*δ*, as compared to the vector-transfected cells (Figure 2(g)).

### 3.3. Overexpression of PPAR*β*/*δ* Suppresses HepG2 Migration and Invasion

**Figure 3:**
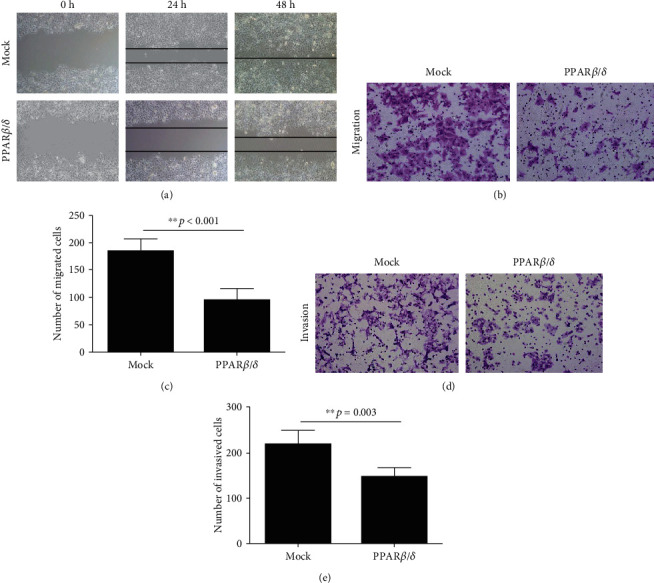
Effect of PPAR*β*/*δ* on liver cancer cell motility and invasion capability, as assessed by wound healing and Matrigel invasion assays. HepG2 cells stably transfected with pEGFP-PPAR*β*/*δ* or the pEGFP vector (control) were subjected to (a) a wound healing assay and (b) a cell migration assay. Representative pictures were taken under an inverted microscope at the indicated time points. (c) Cell motility was quantified by counting the cells that migrated through the Matrigel membrane under a light microscope (×100). The relative cell number ratio was expressed as the mean ± standard deviation. ∗∗*p* < 0.001, compared to the control. (d) Representative images of the cell invasion ability of HepG2 cells transfected with pEGFP-PPAR*β*/*δ* or the control vector after 48 h. (e) Quantification of cell invasion was estimated by counting the cells that invaded through the Matrigel membrane under a light microscope (×100). The data are expressed as the mean ± standard deviation. ^∗∗^*p* < 0.01, compared to the control.

Wound-healing assays were conducted to evaluate migration in PPAR*β*/*δ*-overexpressed HepG2 cells. As shown in Figure 3(a), HepG2_mock cells spontaneously migrated and filled the wounded area within 48 h, while the migration of HepG2_PPAR*β*/*δ* cells was blocked or inhibited even after 48 h. In accordance with the results observed in the scratch assays, elevated expression of PPAR*β*/*δ* markedly attenuated the migration (*p* < 0.05, Figures 3(b) and 3(c)) and invasion of HepG2 cells (*p* < 0.05, Figures 3(d) and 3(e)) in the transwell migration and invasion assays. Taken together, these results indicate that PPAR*β*/*δ* is a potent suppressor of hepatoma cell migration and invasion.

### 3.4. PPAR*β*/*δ* Modulates the Expression Profiles of Cancer-Related Genes in HepG2 Cells

**Figure 4:**
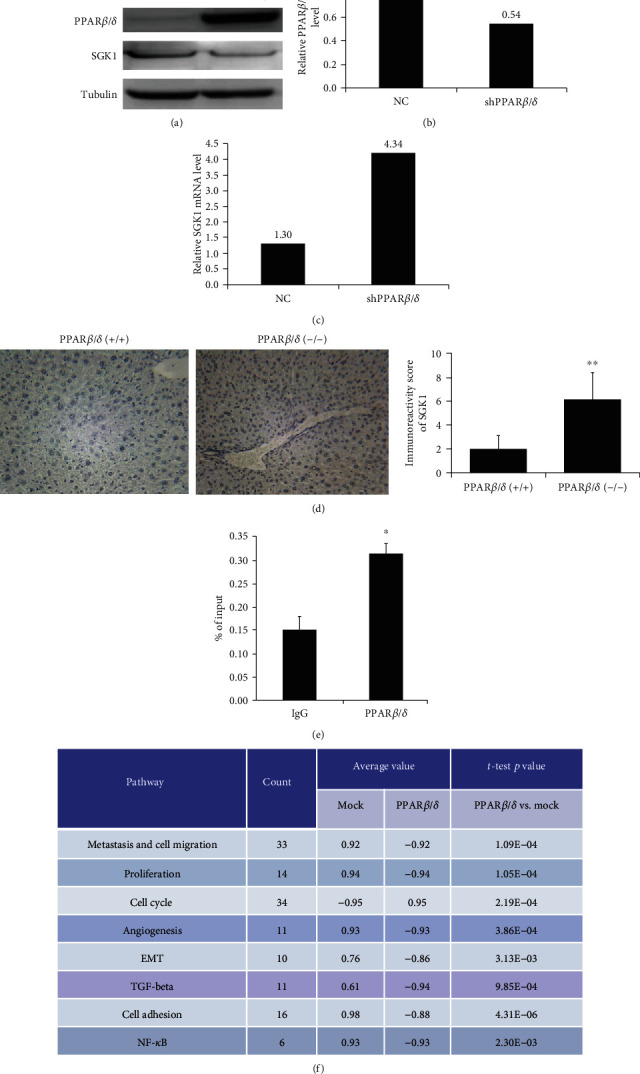
PPAR*β*/*δ* regulates the expression of SGK-1. (a) Western blot analysis of SGK-1 in PPAR*β*/*δ*-overexpressed HepG2 cells. (b, c) The mRNA levels of PPAR*β*/*δ* and SGK-1 were determined in shPPAR*β*/*δ* cells using qPCR, respectively. (d) Representative images of immunohistochemical staining from PPAR*β*/*δ*^(-/-)^ mice and control mice, with a higher expression of SGK-1 in PPAR*β*/*δ*^(-/-)^ mice. (e) ChIP-qPCR assays confirmed that the transcription factor PPAR*β*/*δ* can specifically bind to the regulatory region of SGK1 in HepG2 cells. Bars correspond to the mean ± standard deviation. ^∗^*p* < 0.05, compared to the isotype-matched IgG control (IgG). (f) Whole-genome microarray analysis of gene expression in HepG2 cells transfected with PPAR*β*/*δ*_pEGFP-N1 or empty vector. Functional annotation was carried out in tabulation.

To elucidate the molecular mechanisms underlying the inhibitory effect of PPAR*β*/*δ* on HCC growth, the gene expression profiles in pEGFP-PPAR*β*/*δ*-transfected HepG2 cells were analyzed using whole-genome expression arrays from Illumina (humanHT-12_v4 beadchips). Principal component analysis utilizing the entire gene expression dataset showed the relatively tight clustering of the two groups and the clear separation of the experimental group from the control group. Compared with mock transfection, 222 upregulated and 382 downregulated genes were found in HepG2_PPAR*β*/*δ* cells. GSEA of the PPAR*β*/*δ* target genes revealed a significant drop in the average expression of genes related to metastasis and cell migration, cell adhesion, proliferation, angiogenesis, epithelial-to-mesenchymal transition, nuclear factor-*κ*B, and transforming growth factor *β* signaling pathways, while upregulation in the average gene expression of cell cycle regulators (Figure 4(f)).

### 3.5. PPAR*β*/*δ* Transcriptionally Downregulates SGK-1 Expression

Expression array analysis indicated a 7.79-fold decrease in the abundance of SGK-1 expression in PPAR*β*/*δ*-overexpressed HepG2 cells. SGK-1 was one of the most downregulated genes. The downregulation of the SGK-1 expression by PPAR*β*/*δ* was confirmed by western blot (Figure 4(a)). The mRNA level of SGK-1 was noticeably increased when the PPAR*β*/*δ* activity was suppressed in SMMC-7721 cells infected with LV008-shPPARD (Figures 4(b) and 4(c)). A higher expression of SGK-1 protein was also detected in the livers of the PPAR*β*/*δ*^(-/-)^ mice compared to that of the wild-type mice by immunohistochemistry (Figure 4(d)). These results indicated that PPAR*β*/*δ* might play a catalytic role through binding to the SGK-1 gene promoter. ChIP assays were performed on pEGFP-PPAR*β*/*δ*- or control vector-transfected HepG2 cells. Primarily, the transcription factor binding sites in the SGK-1 regulatory regions were evaluated using the JASPAR database (http://jaspar.genereg.net/cgi-bin/jaspar_db.pl), and the PPAR*β*/*δ* recognition site (CCAGGCTAAAGTGCA) was found in the 5′-regulatory region of the SGK-1 gene, which points to the role of the transcription factor PPAR*β*/*δ* in the expression of SGK-1. The immunoprecipitation was performed using an anti-PPAR*β*/*δ* antibody in chromatin DNA fragments, and a 163 bp fragment of the SGK-1 sequence was amplified from the immunoprecipitated DNA, indicating the direct binding of PPAR*β*/*δ* to SGK-1 (Figure 4(e)).

## 4. Discussion

Over the past decade, many studies have revealed the health benefits of PPAR*β*/*δ* in combating inflammation, lipogenesis, and insulin resistance. Activation of PPAR*β*/*δ* has been shown to have anticarcinogenic effects in skin cancer [24], pancreatic cancer [19], and prostate cancer [18], albeit not without controversy [15]. The role of PPAR*β*/*δ* in liver tumorigenesis has been established as well. Using a DEN-induced murine model of HCC, we demonstrated that a lack of PPAR*β*/*δ* increased the susceptibility to HCC formation. Our results were consistent with other studies using PPAR*β*/*δ*-knockout mice that showed an increased incidence of skin cancer [21], larger intestinal tumors [25], and chemically induced liver toxicity [23]. In addition, it has been reported that PPAR*β*/*δ* has an antiproliferative influence on prostate cancer cells, keratinocytes, and melanoma cells [24, 26, 27]. In order to investigate the effect of endogenous transactivation of PPAR*β*/*δ* in liver carcinogenesis, we examined its functional consequences by overexpressing PPAR*β*/*δ* in human HepG2 liver cancer cells. We found that the overexpression of PPAR*β*/*δ* resulted in inhibition of HepG2 cell proliferation in a time-dependent manner. The subsequent Hoechst staining and flow cytometry assays revealed that PPAR*β*/*δ* could induce apoptotic cell death and cell cycle arrest. Consistently, Coleman et al. have demonstrated that PPAR*β*/*δ* activation prevents the invasion and migration abilities of pancreatic cancer cells by activating the B cell lymphoma 6 pathway [19, 28]. Moreover, the current study revealed that overexpression of PPAR*β*/*δ* inhibited the liver cancer cell migration and invasion abilities.

It is well established that PPAR*β*/*δ* plays an important role in lipid and glucose metabolism and that it could be a potential molecule that links metabolism and carcinogenesis. The current study demonstrated by microarray analysis that SGK1, a member of the protein kinase A, G, and C families, is downregulated by PPAR*β*/*δ*. The immunohistochemistry results also supported this observation as the SGK-1 level was higher in PPAR*β*/*δ*^−/−^ mice. Previous data have shown that PPAR*γ* agonists induce the SGK-1 gene expression by direct binding [29]. The current study is the first to show that PPAR*β*/*δ* also regulates the SGK-1 gene expression but in a negative way. SGK-1 transcription is stimulated by excessive glucose levels and diabetes, oxidative stress, DNA damage, ischemia, neuronal injury, and a high-fat diet [30–33]. In addition, active SGK-1 induces insulin release, adipocyte differentiation, and adipogenesis [31, 34]. The Human Protein Atlas database also shows elevated SGK-1 levels in liver cancer, colon cancer, myeloma, medulloblastoma, prostate cancer, ovarian tumors, and non-small-cell lung cancer [35]. Moreover, SGK-1-knockout mice are resistant to chemically induced colon carcinogenesis [31]. Recent findings also have shown that SGK-1 regulates cell survival, proliferation, and differentiation in several types of cancer cells such as kidney [31], breast [36], and liver cancer [37]. Additionally, SGK-1 may promote the survival of cholangiocarcinoma cells by mediating the IL-6-related pathway [38]. Furthermore, angiotensin II protects fibrosarcoma-derived cells from apoptosis by increasing SGK-1 phosphorylation [39]. Meanwhile, activated PPAR*β*/*δ* prevents IL-6-induced insulin resistance by inhibiting the signal transducer and activator of transcription 3 pathway in adipocytes, which was enhanced in PPAR*β*/*δ*-null mice [10]. Another study has suggested that PPAR*β*/*δ* protects against lipid accumulation and oxidative stress by reducing angiotensin II-induced activation of the Wnt signaling pathway [40]. Thus, through different signaling pathways, PPAR*β*/*δ* is implicated in metabolism and growth.

## 5. Conclusions

In conclusion, our data suggest that PPAR*β*/*δ* is a tumor suppressor in HCC and that downregulation of SGK-1 may be implicated in its tumor-suppressive effect.

## Data Availability

The datasets generated and analyzed during the present study are available from the corresponding author on reasonable request.
